# The role of expectations, control and reward in the development of pain persistence based on a unified model

**DOI:** 10.7554/eLife.81795

**Published:** 2023-03-27

**Authors:** Christian Büchel

**Affiliations:** 1 Department of Systems Neuroscience, University Medical Center Hamburg-Eppendorf Hamburg Germany; Maastricht University Netherlands; University of Michigan United States

**Keywords:** chronic pain, pain, brain

## Abstract

Chronic, or persistent pain affects more than 10% of adults in the general population. This makes it one of the major physical and mental health care problems. Although pain is an important acute warning signal that allows the organism to take action before tissue damage occurs, it can become persistent and its role as a warning signal thereby inadequate. Although per definition, pain can only be labeled as persistent after 3 months, the trajectory from acute to persistent pain is likely to be determined very early and might even start at the time of injury. The biopsychosocial model has revolutionized our understanding of chronic pain and paved the way for psychological treatments for persistent pain, which routinely outperform other forms of treatment. This suggests that psychological processes could also be important in shaping the very early trajectory from acute to persistent pain and that targeting these processes could prevent the development of persistent pain. In this review, we develop an integrative model and suggest novel interventions during early pain trajectories, based on predictions from this model.

## Psychological and biological models of pain

The field of pain has developed elegant models for pain perception and pain trajectories. The biologically informed gate control theory (Melzack and Wall, 1965) posits a spinal gate that is under the influence of a ‘central control’ (and other factors), and indirectly arguing for a psychological influence. The motivation-decision model (Fields, 2006; Fields, 2018) and related models (Wiech et al., 2014) extend this view and add a decision component between pain-related behaviors and competing motivational states. This model assumes that there is a constant decision process, in which the organism has to decide whether it is advantageous to attend to pain, or an alternative motivational state. Both theories make biological predictions with respect to the activation of the descending pain modulatory system (DPMS) including the brain, brainstem and spinal cord (Ren and Dubner, 2009; Heinricher and Fields, 2013). Additionally, there are recent psychological expectation models (Büchel et al., 2014; Rief and Petrie, 2016; Wiech, 2016; Ongaro and Kaptchuk, 2019), which posit that pain is the result of a (Bayesian) integration of expectation with nociception. The fear-avoidance model (Vlaeyen et al., 1995) explains how negative expectations lead to a lack of agency, which is seen as a crucial step in pain persistence. Finally, the learned helplessness model (Maier and Seligman, 1976), originally developed in the context of stress and depression, posits that when being exposed to repeated aversive stimuli (e.g. pain) that are out of control, the individual does not attempt to escape or avoid the aversive stimulus. This model has gained momentum in pain, as it has been shown that helplessness is strongly related to chronic pain (Samwel et al., 2006). Unfortunately, the links between the models are sparse, which is an important limitation, as it is widely accepted that pain is best understood in the context of a holistic model (Engel, 1977; Gatchel et al., 2007). We acknowledge that social aspects play a crucial role in pain trajectories, but for reasons of brevity, this review will only focus on psychobiological aspects.

### An integrative model

**Figure 1.**
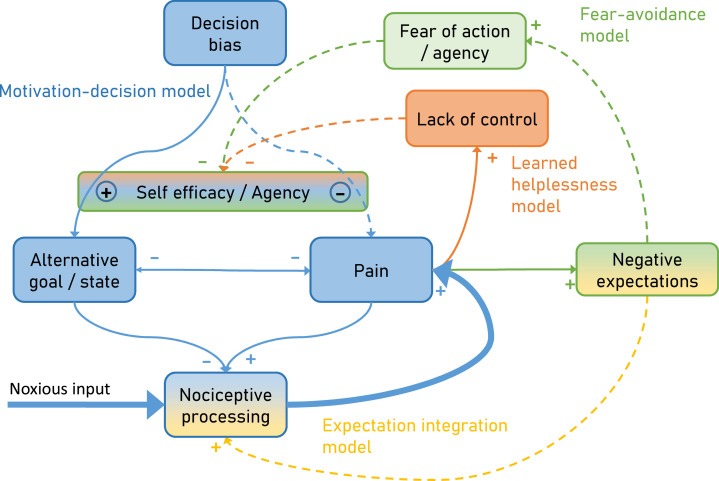
Integrative psychobiological pain model, incorporating the motivation-decision model (blue) (Fields, 2006), the fear-avoidance model (green) (Vlaeyen et al., 1995), learned helplessness (orange) (Maier and Seligman, 1976) and a Bayesian expectation integration model (yellow) (Büchel et al., 2014). The state of the model depicts the situation for acute pain, with a major noxious input that generates pain via nociceptive processing. In addition, acute pain already generates negative expectations and some loss of control. Dashed arrows depict minor contributions, solid arrows depict medium contributions and bold arrows strong contributions.

**Figure 2.**
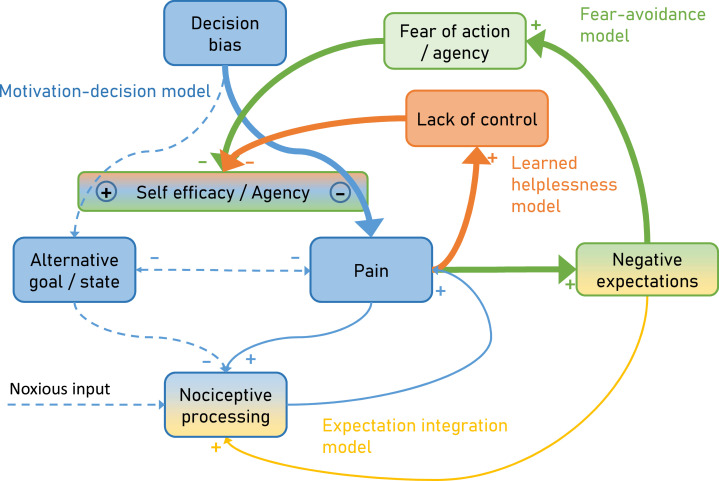
Over time and with ongoing pain persistence, the influence of nociceptive processing on pain gets weaker and at the same time, pain is maintained by fear of agency (Fear-avoidance model) and lack of control (Learned helplessness model). Importantly, the model suggests that both aspects act through the arbitrator of the Motivation-decision model. In addition, negative expectations can increase pain through integration with nociceptive input. Dashed arrows depict minor contributions, solid arrows depict medium contributions and bold arrows strong contributions.

To overcome this limitation, we developed an integrative model (Figure 1) explaining pain by combining the motivation-decision model (blue) (Fields, 2006), the fear-avoidance model (green) (Vlaeyen et al., 1995), learned helplessness (orange) (Maier and Seligman, 1976) and a Bayesian expectation integration model (amber) (Büchel et al., 2014) and follow up on earlier integrative models (Ingvar, 2015; Borsook et al., 2018). Importantly, the presented model has a dimension of time, which allows it to capture the dynamic nature of pain persistence and makes explicit predictions about its temporal development (Figure 2). Although here the model is setup to account for ‘pain’, alternative formulations of the model could be developed to account for ‘interference with daily life’ or ‘disability’.

In the motivation-decision model, there is a constant decision process in acute pain (Fields, 2006). The organism decides (subconsciously or consciously) whether it is advantageous to attend to pain, or a competing motivational state (Leknes and Tracey, 2008). As in value-based decision making, this decision process is heavily dependent on expectations (Wiech et al., 2014) or attention (Krajbich et al., 2010). Acute pain is an important signal for imminent tissue damage and usually entails an action sequence, targeted at limiting or stopping the pain. However, if over long periods of time pain cannot be controlled (Crombez et al., 2012), a lack of self-efficacy and control emerges, which leads to a state of helplessness (Maier and Seligman, 1976). Self-efficacy is an important cognitive factor in the control of pain (Bandura et al., 1987) and it has been shown that it can lessen experienced pain by shifting priorities away from pain attention to competing goals (Figure 1 orange; Bandura et al., 1987).

Saliency, which drives attention, has been established as a factor in pain and decision making (Litt et al., 2011; Mouraux et al., 2011; Baliki and Apkarian, 2015; Horing et al., 2019). Given that pain often has a higher saliency than competing positive behavioral motives (Mouraux et al., 2011), a further failure to attend to these alternative motives can enhance this effect, for instance by deficient self-efficacy (as in learned helplessness). This directly links learned helplessness with the motivation-decision model (Figure 1 blue), arguing that helplessness will entail a failure to shift attention away from pain, towards a positive alternative behavioral goal. Finally, in the fear-avoidance model (Lethem et al., 1983; Vlaeyen et al., 1995; Meulders, 2019) fear of pain and negative expectations lead to fear of action and this again reduces self-efficacy (Figure 1 green). The fear-avoidance model highlights that catastrophic appraisals of pain experience can be carried over via negative expectations including fear (Vlaeyen and Linton, 2000; Boersma and Linton, 2006). Interestingly, some have argued that the fear-avoidance model can increase its explanatory power by taking the motivational perspective into account, namely that patients show unwillingness to pursue valued activities, marking a formal link to the motivation-decision model (Crombez et al., 2012; Claes et al., 2015). However, even in a smaller control loop (Figure 1 amber), nociception, pain and negative expectations can represent a vicious circle: It has been demonstrated that negative expectancies are produced by frequent pain, and that more frequent and more intense pain leads to more pain-related fear and more expectations of persistent pain (Boersma and Linton, 2006; Fields, 2018). Importantly, negative expectations of persistent pain and beliefs of fear-avoidance have a unique predictive value for future pain and disability (Boersma and Linton, 2006). This could be relevant in view of the notion that nociception at a subthreshold level is an ongoing process, which only when crossing a threshold becomes pain (Baliki and Apkarian, 2015; Apkarian, 2019) and thus is ever present. This further emphasizes the importance of this loop in pain and possibly in pain persistence.

This model explicitly links more biologically oriented and more psychologically oriented models. In particular, it suggests that the consequences of fear of actions (fear avoidance model) affect the arbitration in the motivation-decision model and thus provides a biological mechanism through which fear avoidance can affect pain. In a similar manner, the model suggests that lack of control as described by the learned helplessness model can also affect the arbitration stage of the motivation-decision model and provides a mechanism of how a lack of agency can influence pain. Furthermore, the expectation integration model is assumed to exert its influence on pain by a gate-control mechanisms, for example at the spinal (Eippert et al., 2009b) or mid-brain level (Grahl et al., 2018).

Key concepts of the proposed model are typical elements of associative learning. For example, negative expectations which are also at the core of Pavlovian conditioning, are proposed to influence (i) nociceptive integration (Expectation-integration model) and (ii) are the basis for fear of action (Fear avoidance model). The latter is especially important as it is suggested that negative expectations that were initially specific to the causing pain can generalize to safe situations or persist longer than needed. This is in agreement with studies showing that excessive negative expectancy, fear and lack of safety identification are observed in chronic pain patients (Harvie et al., 2017).

Furthermore, the proposed model incorporates the dynamic aspects of pain persistence and makes predictions about the temporal development of pain persistence (Figure 2). Over time and with ongoing pain, negative expectations increase. These increased negative expectations can affect pain through two pathways. First it increases pain through the fear of agency pathway (Fear-avoidance model; Figure 2 green), which acts by tipping the arbitrator of the Motivation-decision model towards pain and away from alternative goals (Figure 2 blue). Secondly, negative expectations can increase pain through a direct integration with nociceptive input (Figure 2 amber) as outlined by the Expectation integration model. Prolonged pain, which is hard to control also starts to generate the perception of a lack of control (Learned helplessness model; Figure 2 orange). This in turn biases the arbitrator of the of the Motivation-decision model towards pain and away from alternative goals.

Consequently, we suggest that expectation, reward, and controllability could be promising targets in the fight for preventing pain persistence and explain how targeting these concepts could prevent pain persistence with explicit links to the presented model. Finally, we propose possible experimental approaches to investigate the derived hypotheses and end with a general perspective on studies in patients and healthy volunteers to investigate the development of pain persistence.

### Expectation

Previous studies Ploghaus et al., 1999; Koyama et al., 2005; Atlas and Wager, 2012; Geuter et al., 2017; Fazeli and Büchel, 2018; Horing and Büchel, 2022 have investigated the effect of expectation on pain perception using behavioral and neurobiological readouts. These effects are also main constituents of placebo (Wager et al., 2004; Bingel et al., 2006; Eippert et al., 2009a; Buhle et al., 2012) and nocebo effects (Scott et al., 2008; Tracey, 2010; Jensen et al., 2012; Geuter and Büchel, 2013; Tinnermann et al., 2017). Many of these studies have identified an important role of the dorsolateral (dlPFC) (Wager et al., 2004; Tétreault et al., 2016; Vachon-Presseau et al., 2018) and medial prefrontal cortex (mPFC) (Bingel et al., 2006; Eippert et al., 2009a; Geuter et al., 2013) in mediating expectation effects. Importantly, the dlPFC has also been implicated in chronic pain (Seminowicz and Moayedi, 2017). In addition, network analyses have revealed that expectation effects are mirrored by changes in coupling in the descending pain modulatory system (Porreca et al., 2002; Fields, 2004; Tracey and Dunckley, 2004), in particular between the mPFC and the periaqueductal gray (PAG) (Bingel et al., 2006; Eippert et al., 2009a; Tinnermann et al., 2017; Drake et al., 2021). Novel ideas of how one’s own (Anchisi and Zanon, 2015; Grahl et al., 2018) or observed pain (Yoshida et al., 2013) results in expectations that are finally integrated with nociceptive information, have led to a Bayesian integration model involving the PAG (Yoshida et al., 2013; Büchel et al., 2014; Grahl et al., 2018). Subsequently, this framework has been extended to expectations in the context of pain processing in general (Ongaro and Kaptchuk, 2019).

Current pain is a reliable predictor for future pain and therefore it is difficult to avoid negative expectations in the context of acute pain (Eccleston et al., 2013; Fields, 2018). According to our model, these negative expectations can support pain persistence via two separate routes. First, negative expectations get integrated with nociception and according to the Bayesian integration part of the model will increase pain (Figure 1 yellow). This integration takes into account the incoming sensory information (nociception) and expectations and more importantly weighs both by their respective precision. Secondly, according to the fear-avoidance part, negative expectations lead to increased fear of action and reduced agency (Figure 1 green), which in turn shifts the balance of the motivation-decision part (Figure 1 blue) of the model towards pain (as opposed to alternative goals). This shows the pivotal role of negative expectations in pain persistence fueling two vicious circles amplifying pain.

One strategy to reduce negative expectations could be to exploit the natural fluctuations which are typical for clinical pain (Baliki et al., 2006; Mayr et al., 2022). Increasing attention to pain decreases and decreasing attention to pain increases could help to reduce pain and thus the ensuing negative expectations. However, it should be noted that pain by virtue of its role in signaling potential tissue damage is highly salient (Mouraux et al., 2011) and thus attention capturing (Eccleston and Crombez, 1999). Nevertheless, reducing attention to pain might be achieved by demanding and engaging concurrent tasks that can compete for attentional resources for example working memory (Sprenger et al., 2012), which has been shown to reduce pain by about 20%. Attentional modulation is a promising intervention as it can easily be implemented and has been shown to effectively interfere with acute pain processing (Sprenger et al., 2012; Kucyi et al., 2013; Brooks et al., 2017; Schulz et al., 2020; Oliva et al., 2022). Attentional shifts away from pain might also be the mechanisms underlying the observation that early return to work has benefits for relief of back pain and functional recovery (Shaw et al., 2018).

### Reward

With regard to reward, rodent and human studies have shown that reward and hedonic experiences can lead to substantial hypoalgesia (de Freitas et al., 2012; Becker et al., 2013; Navratilova et al., 2016; Porreca and Navratilova, 2017), mediated by the release of endogenous opioids in the descending pain modulatory system (DPMS). Nevertheless, these effects have mainly been established in acute pain experiments and there is less knowledge about the effect of reward on long-term pain (Leknes and Tracey, 2008; Benedetti et al., 2013).

Studying the effects of reward on pain persistence seems promising, because previous studies have suggested changes in the reward system in chronic pain patients (Baliki et al., 2012; Navratilova et al., 2016) and that chronic pain patients exhibit poorer reward responsiveness (Elvemo et al., 2015). The neurotransmitter dopamine plays a pivotal role in reward processing and therefore it is not surprising that the mesocorticolimbic reward system might be disturbed in chronic pain (Letzen et al., 2019) and that the baseline dopamine metabolism is reduced in chronic back pain patients (Martikainen et al., 2015).

Reward-related processes might also be the mechanisms underlying the observation that functional connectivity between the ventral striatum and the medial frontal cortex is associated with chronification in back pain patients (Baliki et al., 2012). In particular, it has been shown that the transition to chronic pain is related to changes in cortico-striatal networks (Apkarian et al., 2013). Furthermore, it has been shown that learning-related updating of the value of reinforcement (prediction error) in the ventral striatum predicts the transition to chronicity (Löffler et al., 2022).

Based on the presented model, we would argue that providing a motivationally salient alternative to pain can change the organisms decision (Figure 1 blue) to engage in alternative behaviors, which according to the motivation-decision part of the model, would also entail an activation of the DPMS, reduce pain and probably reduce the risk that patients will develop chronic pain (Price et al., 2018).

This would require that acute pain is combined with a rewarding alternative, which is salient enough to tip the motivation-decision arbitrator towards pursuing the reward (Figure 1 blue) and overcome the ‘stickiness’ of pain (Borsook et al., 2018). A possible experimental task could be a motivationally engaging foraging task with salient primary (e.g. food) or secondary (e.g. money) reinforcers. Such an intervention would also reduce the attentional resources for the painful stimulus (see above) and thus additionally reduce pain by shifts of attention. Evidence from a study on acute pain, in which participants had to decide between accepting a reward at the cost of receiving pain, showed that accepting a reward (especially one with a high subjective utility) coupled to a nociceptive stimulus resulted in decreased perceived intensity (Becker et al., 2017). In general, the value of reinforcers heavily depends on the state of the organism (i.e. low value of food when satiated) and this is expected to modulate the effect of reward on pain.

### Controllability

It has been shown that perceived control affects pain tolerance, learning and motivation, and the ability to cope with intractable pain (Bandura et al., 1987). This has been corroborated in a recent study by showing that controllability can reduce the experience of pain-related suffering (Löffler et al., 2018). Perceived control of pain has been shown to be associated with increased activation in the mPFC and reduced activation in the anterior cingulate, insular, and secondary somatosensory cortex (Salomons et al., 2004) and is associated with individual differences in the structure of the central motor output system (Salomons et al., 2012). In addition, a pain-facilitating role of the mPFC during uncontrollable pain and a pain-inhibiting role of the dlPFC during controllable pain was identified, both exerting their respective effects via the anterior insula (Bräscher et al., 2016). Again, this has mainly been studied in the context of acute or short-term pain paradigms, but is of importance for chronic pain (Bushnell et al., 2013; Drake et al., 2021).

Based on these findings, a possible intervention to block pain persistence would be to increase controllability of pain. This seems impossible, given that acute, and especially chronic pain are characterized by the absence of control (Bushnell et al., 2013). However, human subjects can perceive control even if their actions and the ensuing outcomes are only weakly coupled, known as the illusion of control (Taylor et al., 1991; Johnson and Fowler, 2011). In a possible experimental approach, one might track individual pain level fluctuations in real-time for example using peripheral signals such as pupil size or electrodermal activity (Geuter et al., 2014). Given that these measures are rather unspecific, it would be desirable to add additional readouts such as facial video recordings (Lautenbacher et al., 2022), kinematics (Turri et al., 2022) and EEG measures (May et al., 2019). Once the beginning of a spontaneous pain decrease is identified, the volunteer or patient will be offered the opportunity to self-initiate a treatment. Although pain is spontaneously decreasing, and not through the putative treatment, the tendency of humans to perceive control, might ‘reverse’ causality and lead to a sense of agency (i.e. control over pain). This could ameliorate the feedback loop of control (Figure 1 orange) with the consequence of biasing the arbitrator of the motivation-decision part (Figure 1 blue) away from pain. Although this might be difficult to implement in pain patients, it is still promising as studies in chronic back pain have shown that increased perceptions of control over pain were related to reductions in disability (Woby et al., 2004).

### General aspects and required studies

As outlined in the previous paragraphs, there is a possibility that manipulating controllability, reward and expectation could alter the trajectory of pain persistence. However, integrating these manipulations into experimental paradigms is not straight forward. A categorical problem of studying the trajectory from acute to persisting pain is the complexity associated with longitudinal studies in patients. In particular, the duration of these studies needs to cover a long period of time and the number of enrolled patients needs to be large, given that only a fraction of acute pain patients develops persistent pain. Nevertheless, there have been heroic studies of this kind, which have brought important insights into the mechanisms and prognostic markers of pain persistence (Baliki et al., 2012; Mansour et al., 2013; Löffler et al., 2022). In addition, studies in chronic pain are difficult to control with respect to nociceptive input, comorbidities, medication and other patient characteristics, which especially in small samples, can decrease sensitivity. Finally, in patients, it might be difficult to capture the very early phase of the pain trajectory, which, as argued in this review, could be crucial for the development of persistent pain (Price and Ray, 2019). This is in agreement with a recent criticism of the definition of chronicity as it is agnostic to the point when the transition from acute to chronic pain occurs (Price and Ray, 2019).

Therefore, we propose that longitudinal studies in patients should be complemented by longitudinal studies (i.e. over several weeks) in healthy subjects, employing precisely controlled daily pain exposure to test for the effects of expectation, reward and controllability. Examples of these studies have indicated that experimentally induced pain over weeks in healthy volunteers can produce neurobiological signs of persistent pain (Apkarian et al., 2004; May, 2008; Bushnell et al., 2013) such as sensitization (Stankewitz et al., 2013) and structural brain changes (May, 2008; Teutsch et al., 2008; Stankewitz et al., 2013) highlighting their potential as a first screening instrument for possible modulators of pain persistence. These studies could be further improved by novel methodology including computational modeling (Seymour, 2019) and neuroimaging (Davis, 2019), including novel developments such as combined brain-spinal cord fMRI (Finsterbusch et al., 2013), to make formal links between biological mechanisms (such as sensitization or shift in excitation-inhibition balance) and psychological constructs (such as negative expectations and loss of control). Once these studies in healthy volunteers have identified promising modulators of pain persistence, larger and well powered clinical trials in acute pain patients (e.g. back pain) could be initiated to test these interventions in a clinical setting.
